# Psychometric evaluation of a Canadian version of the Seattle Angina Questionnaire (SAQ-CAN)

**DOI:** 10.1186/s12955-020-01627-2

**Published:** 2020-12-01

**Authors:** Oluwaseyi A. Lawal, Oluwagbohunmi Awosoga, Maria J. Santana, Matthew T. James, Danielle A. Southern, Stephen B. Wilton, Michelle M. Graham, Merrill Knudtson, Mingshan Lu, Hude Quan, William A. Ghali, Colleen M. Norris, Tolulope Sajobi, for the APPROACH Investigators

**Affiliations:** 1 Department of Community Health Sciences, University of Calgary, 3280 Hospital Drive NW, Calgary, AB Canada; 2 Faculty of Health Sciences, University of Lethbridge, Lethbridge, Canada; 3 Department of Cardiac Sciences, University of Calgary, Calgary, Canada; 4 Faculty of Medicine and Dentistry, University of Alberta, Edmonton, Canada; 5 Department of Economics, University of Calgary, Calgary, Canada; 6 Faculty of Nursing, University of Alberta, Edmonton, Canada

**Keywords:** Seattle Angina Questionnaire, Psychometric properties, Heart disease, Canadian, Stable angina, Health-related quality of life

## Abstract

**Background:**

The Seattle Angina Questionnaire (SAQ) is a widely-used patient-reported outcomes measure in patients with heart disease. This study assesses the validity and reliability of the SAQ in a Canadian cohort of individuals with stable angina.

**Methods and results:**

Data are from the Alberta Provincial Project for Outcome Assessment in Coronary Heart Disease (APPROACH) registry, a population-based registry of patients who received cardiac catheterization in Alberta, Canada. The cohort consists of 4052 patients undergoing cardiac catheterization for stable angina and completed the SAQ within 2 weeks. Exploratory factor analysis and confirmatory factor analysis (CFA) were used to assess the factorial structure of the SAQ. Internal and test–retest reliabilities of a new measure (i.e., SAQ-CAN) was measured using Cronbach *α* and intraclass correlation coefficient, respectively. CFA model fit was assessed using the root mean square error of approximation (RMSEA) and comparative fit index (CFI). Construct validity of the SAQ-CAN was assessed in relation to Hospital Anxiety and Depression Scales (HADS), Euro Quality of life 5 dimension (EQ5D), and original SAQ. Of the 4052 patients included in this analysis, 3281 (80.97%) were younger than 75 years old, while 3239 (79.94%) were male. Both exploratory and confirmatory factor analyses revealed a four-factorial structure consisting of 16 items that provided a better fit to the data (RMSEA = 0.049 [90% CI = (0.047, 0.052)]; CFI = 0.975). The 16-item SAQ demonstrated good to excellent internal reliability (Cronbach’s α range from 0.77 to 0.90), moderate to strong correlation with the Original SAQ and EQ5D but negligible correlations with HADS.

**Conclusion:**

The SAQ-CAN has acceptable psychometric properties that are comparable to the original SAQ. We recommend its use for assessing coronary health outcomes in Canadian patients with Coronary Artery Disease.

## Introduction

Heart disease, the second-leading cause of death in Canada, affects up to 8.5% [1] of adult Canadians and accounts for an annual estimated cost of $21.2 billion [2]. In addition to conferring increased risks of premature mortality and major non-fatal morbidity, chronic heart disease leads to significant ongoing symptoms and associated impairment in functional status and health-related quality of life (HRQOL) [3]. Professional societies, such as the American Heart Association, have advocated integrating patients’ perspectives of their health status as a key cardiovascular health outcome that should be used in clinical trials of new interventions, observational studies, and routine clinical practice [4]. Consequently, the assessment of the HRQOL as a primary or secondary outcome in clinical trials and observations studies of patients with coronary artery disease (CAD) has continued to grow [4–6].

Several patient-reported outcome measures have been specifically developed for measuring symptoms burden, functional status, and quality of life in people with CAD. The Seattle Angina Questionnaire (SAQ), a widely used disease-specific measure of quality of life in patients with heart disease, is a 19-item self-administered questionnaire that measures 5 dimensions of HRQOL [7]. Originally developed in a population of US veterans, SAQ has been translated into more than 52 languages [8] and adapted for use in several countries. While a number of studies have investigated the construct validity and reliability of the SAQ, only a few studies have examined its factorial validity. In those studies, the original factorial structure of the SAQ was not tenable. For example, Kimble et al.’s validation of the SAQ in a population of predominantly female sample of stable angina patients showed the emergence of new subscales (e.g., division of the physical limitation subscale into two separate factors) and misfit of one of the SAQ items [9]. Similarly, the translation and validation of the Farsi version of the SAQ yielded a five-factor solution with subscales that were not identical to the original SAQ subscales [10]. Garrath et al. examined the psychometric properties of the SAQ in a United Kingdom sample of stable angina patients and found that the original factorial structure of the SAQ resulted in the removal of 4 items, resulting in the emergence of the 15-item United Kingdom version of the SAQ with 3 subscales [11].

Despite its wide use, SAQ has not been previously validated in a Canadian sample of individuals with heart disease. The study investigated the measurement properties of the SAQ in Canadian patients by assessing the validity and reliability of the SAQ in a Canadian cohort of stable angina patients.

## Methods

### Data source

De-identified secondary data were obtained from the Alberta Provincial Project for Outcome Assessment in Coronary Heart Disease (APPROACH) registry [12, 13], a population-based registry of all patients, who had cardiac catheterization in the province of Alberta, Canada. The APPROACH registry contains detailed demographics and clinical information. Individuals in the registry are followed longitudinally after catheterization for assessment of subsequent procedures and patient-reported health status from those who consent to follow up. The study cohort consisted of adults (≥ 18 years) patients with coronary artery disease who underwent cardiac catheterization for stable angina from January 1, 2003, and December 31, 2016, and completed the SAQ 2 weeks after cardiac catheterization. Data collected at catheterization included demographic characteristics (sex, age, address), clinical comorbidities, disease severity measures, and coronary angiography results. Participants also completed several patient-reported outcome measures (PROMs), including the Seattle Angina Questionnaire (SAQ), EuroQol-5-Dimension, Hospital Anxiety and Depression scale (HADS).

### Measures

#### Seattle Angina Questionnaire

The SAQ is a 19-item self-administered questionnaire that measures 5 dimensions of HRQOL for CAD assessed over the past 4 weeks. These include physical limitation (9 Items), angina stability (1 item), angina frequency (2 items) treatment satisfaction (4 items), and disease perception (3 items). The items are scored on a 5-or 6-point Likert scales, and the sum of item scores in each domain is then transformed to scores ranging from 0 (no functioning) to 100 (highest level of functioning) by subtracting the lowest possible score, dividing by the range of the scale and multiplying by 100 [7].

#### EuroQoL-5 dimension

The euro quality of life 5 dimension (EQ5D) is a 5-item generic measure of HRQOL. It has a five descriptive system, including mobility, self-care, usual activities, pain/discomfort, and anxiety/depression. Each item is based on a 3-response Likert scale with response options ranging from “no problem” to “severe problems” [14]. The 5-item scale is also accompanied by a visual analogue scale (VAS) where respondents evaluate the state of their health by indicating a position on a vertical, calibrated line starting at 0 (the worst health state imaginable) to 100 (the best health state imaginable). The EQ5D has been validated in cardiac patients and in several populations (including the Canadian population) and is known to demonstrate good psychometric properties [15–17].

#### Hospital anxiety and depression scale

The hospital anxiety and depression scale (HADS) is a self-reported measure of anxiety and depression. Of the 14 items, 7 items are related to anxiety symptoms, while the other 7 items are related to depressive symptoms. Each is rated on a 4-point Likert scale [18]. The total HADS score ranges between 0 and 42, with 0 to 14 considered as low, 15 to 28 considered as moderate and 29 to 42 considered as high. For each of the anxiety and depression subscales, the scores range between 0 and 21, where 0–7 is considered as low, 8–14 moderate and 15–21 as high. HADS has undergone extensive reliability and validity testing in cohorts with different chronic medical conditions and populations (including Canadian populations) and has been widely validated in patients with heart disease [19, 20]. For this analysis, the baseline information of HADS with a total for each of the subscales was used.

### Statistical analysis

Means and standard deviation (SD) were used to summarize continuous outcomes, while frequencies and percentages were used to summarize categorical variables. The 19 items of the SAQ were assessed for floor and ceiling effects [21]. Exploratory factor analysis (EFA) using the varimax rotation and maximum likelihood extraction method was used to identify the underlying dimensions of the 19 items data [22]. The number of factors was evaluated using the Eigen value criteria (number of Eigen values > 1) and scree plot. Items with component loadings ≥ 0.40 [22] on the dimensions were retained. Confirmatory factor analysis (CFA) was used to test the hypothesis about the optimal factorial structure for the data. Model fit was assessed using the root mean square error of approximation (RMSEA; RMSEA < 0.08) [23] and comparative fit index (CFI; CFI ≥ 0.95) [24]. The item-total correlation as an indicator of item specificity was calculated for the individual items and the sum of the scores on the remaining items in that scale and was considered for both the original SAQ and SAQ-CAN. The internal consistency reliability of the dimensions was assessed using Cronbach’s alpha (α), with 0.7 ≤ α < 0.8 considered acceptable [25]. The reproducibility of the SAQ-CAN and original SAQ using the intra-class correlation coefficient was assessed. Higher intra-class correlation coefficients (range 0–1) indicate greater reproducibility. The responsiveness of the SAQ-CAN was measured using the standardized response mean (SRM) [26] over 1 year. The construct validity of the identified SAQ-CAN dimensions was assessed in relation to validated scales using correlation analysis. Specifically, the association between the identified dimensions and original SAQ-19, HADS_A, HADS_D, and EQ5D using Pearson correlation. The polyserial correlation was used to assess the association between the identified dimensions and the ordinal items of the EQ5D. All analyses were conducted in SPSS and AMOS v25.

## Results

### Descriptive analyses

Table 1 describes the demographic and clinical characteristics of this cohort. Of the 4052 patients included, 3239(79.94%) were male, 771(19.03%) were at least 75 years of age, while 2645(65.28%) had a left ventricular ejection fraction of greater than or equal to 50%. Although this cohort’s demographic characteristics are comparable to the Canadian population of individuals with heart disease [27], this cohort had fewer female patients than the general population of Canadian older adults and seniors [28]. Of the 19 SAQ items, only four items had floor effects above 15%, while most of the items had substantial ceiling effects (see Table 2).

**Table 1:** Baseline characteristics of study participants (N = 4052)

Patients’ characteristics	n (%)
Age, y, mean (SD)	65.93 (9.84)
Sex (male), n (%)	3239 (79.94)
*Left ventricular ejection fraction, n (%)*	
> 50%	2645 (65.28)
35–50%	475 (11.72)
20–34%	110 (2.71)
< 20%	24 (0.59)
Not done	798 (19.69)
HADS-depression, mean (SD)	14.44 (2.93)
HADS-anxiety, mean (SD)	17.04 (4.26)
Diabetes mellitus, n (%)	1106 (27.30)
Hypertension, n (%)	3124 (77.10)
Hyperlipidemia, n (%)	3285 (81.07)
Prior MI, n(%)	647 (15.97)
Prior thrombolytic therapy, n (%)	5 (0.12)
BMI, mean (SD)	30.39 (44.18)
Smoking, n (%)	445 (11.31)
*SAQ subscales, mean (SD)*	
Physical limitation	60.93 (20.75)
Angina stability	71.00 (29.64)
Angina frequency	78.96 (23.18)
Treatment satisfaction	82.21 (17.31)
Disease perception	61.39 (25.25)

NB: Data are presented as frequencies (n or N), percentages (%), standard deviation (SD), HADS: anxiety and depression scale, *MI* myocardial infarction, *BMI* body mass index, *SAQ* Seattle Angina Questionnaire, *SD* standard deviation

**Table 2:** Floor, ceiling item ceiling effect and item-total correlation for SAQ-CAN items

Scale/item	%floor	%ceiling	Item-total correlation
**Indoor physical functioning**			
Dressing	0.22	1.60	0.645
Walking indoors	0.54	2.67	0.560
Showering	0.37	1.90	0.666
**Outdoor physical functioning**			
Walking more than a block	11.60	7.38	0.704
Running or jogging	**27.67**	**29.34**	0.856
Lifting or moving heavy objects	**16.86**	**16.78**	0.808
Participate in strenuous sports	**27.81**	**35.24**	0.809
**Treatment experience**			
Satisfaction that everything being done	1.83	**55.92**	0.790
Satisfaction with doctor’s explanation	1.80	**55.73**	0.735
Overall satisfaction with treatment	1.85	**51.18**	0.842
**Angina symptoms burden**			
Symptoms of angina during strenuous activities	4.00	4.058	0.545
Frequency of symptoms	3.63	**37.02**	0.672
Frequency of use of medication	1.11	**74.56**	0.462
Interference with enjoyment of life?	3.53	**37.29**	0.716
Feelings about symptoms persistent	**23.25**	**22.98**	0.648
Worry about heart attach/death	2.47	**15.72**	0.481

NB:* SAQ-CAN* Seattle Angina Questionnaire Canadian version; The text in bold represents the factor loading names, while bold values indicate item floor and ceiling effects above 15%

### Psychometric analyses

**Fig. 1:**
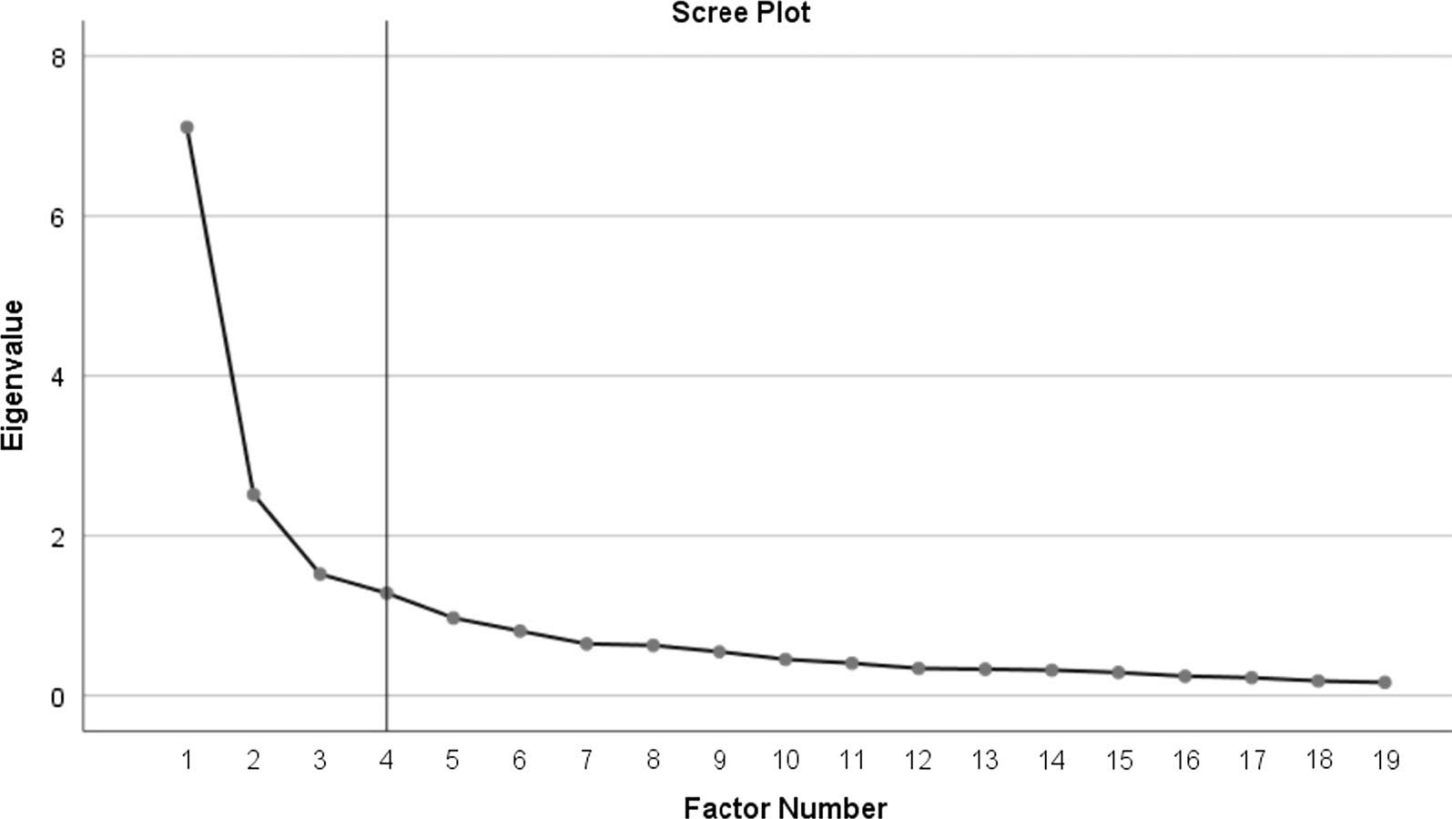
Exploratory factor analysis scree plot for 19-item Seattle Angina Questionnaire

Table 3 describes the results of the EFA, which revealed 4 main dimensions with Eigen values > 1, explaining 57.62% of the variation in the items. A repeat of the EFA without three items; one with smaller factor loadings (“bothersome with taking pills as prescribed”) and two with cross-loadings on two different factors (“climbing a hill/stairs”, “gardening/vacuuming”) resulted in the same 4 dimensions with 60.39% of the variance explained by the factors (See Fig. 1).

**Table 3:** Exploratory factor analysis of SAQ before removal of failed Items

Exploratory factor analysis of the SAQ hypothesised scale/item	Component/factor loadings			
	Factor 1	Factor 2	Factor 3	Factor 4
Dressing	0.733			
Walking indoors	0.612			
Showering	0.757			
**Climbing a hill/stair**	**0.447**	**0.522**	**0.054**	**0.418**
**Gardening/vacuuming**	**0.459**	**0.560**	**0.035**	**0.349**
Walking more than a block		0.652		
Running or jogging		0.881		
Lifting or moving heavy objects		0.793		
Participate in strenuous sports		0.854		
Symptoms of Angina during strenuous activities				0.523
Frequency of symptoms				0.657
Frequency of use of medication				0.482
**Bothersome with taking pills as prescribed**				**0.205**
Satisfaction that everything being done			0.794	
Satisfaction with doctor’s explanation			0.757	
Overall satisfaction with treatment			0.882	
Interference with enjoyment of life?				0.714
Feelings about symptoms persistent				0.692
Worry about heart attach/death				0.492

NB:* SAQ* Seattle Angina Questionnaire. Factor 1: indoor physical functioning, Factor 2: outdoor physical functioning, Factor 3: treatment experience, Factor 4: angina symptoms burden. Bold text and values are items with cross factor loadings and loadings lower than 0.4

**Fig. 2:**
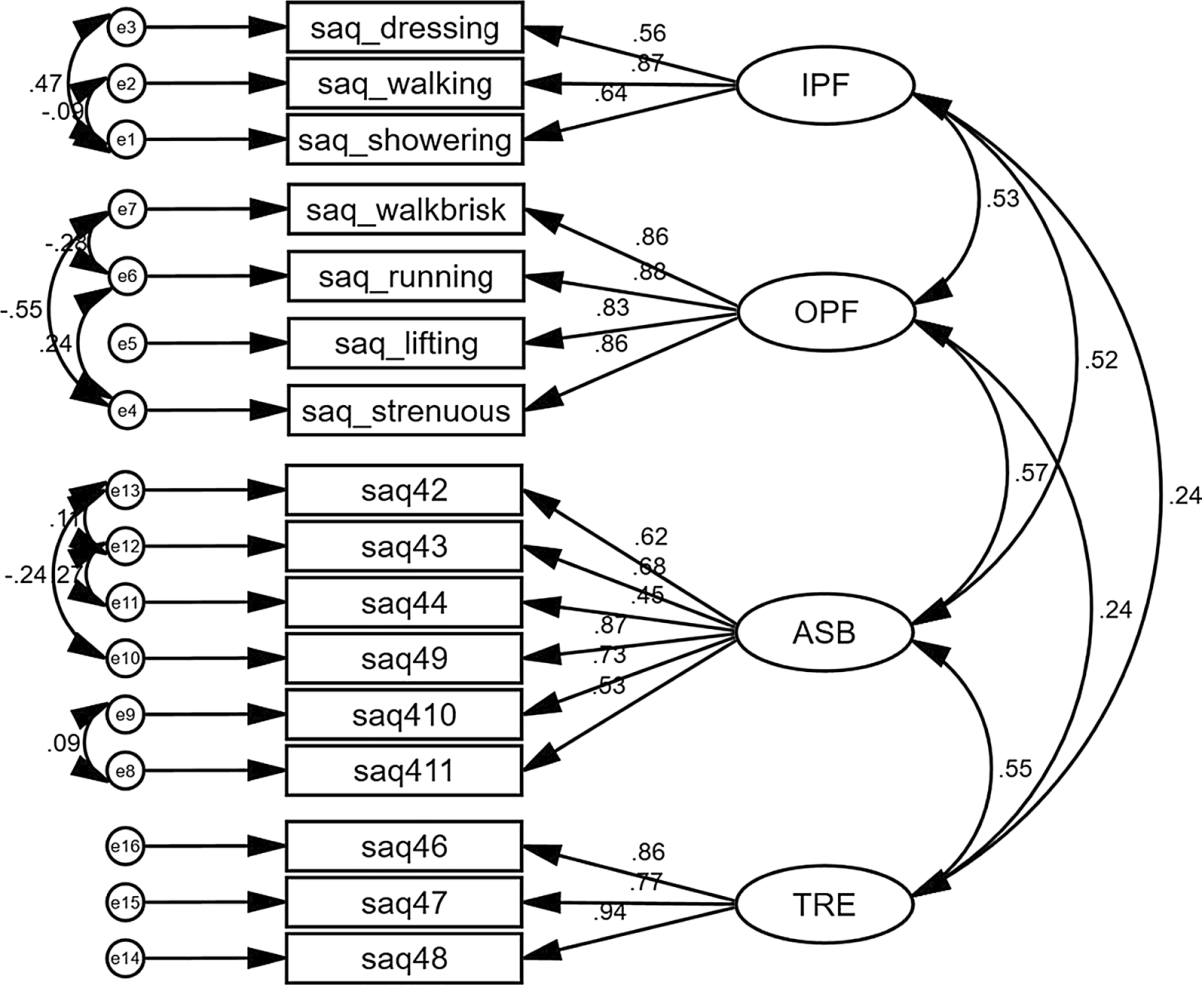
Factorial structure of the Seattle Angina Questionnaire for stable angina. NB: *OPF* outdoor physical functioning, *IPF* indoor physical functioning, *ASB* angina symptoms burden, *TRE* treatment experience

The themes for these four dimensions were indoor physical functioning (3 items), outdoor physical functioning (4 items), treatment experience (3 items), and angina symptoms burden (6 items). Table 4 and Fig. 2 describe the results of the confirmatory factor analysis showing that a four-factorial structure for 16 items provided the best fit for the data (RMSEA = 0.049 (90% CI = [0.047–0.052]) and CFI = 0.975). The factor analytic output displayed in Fig. 2 shows the correlations among measured variables, latent factors of the constructs, and error terms for the variables.

**Table 4:** Exploratory factor analysis of the SAQ for stable angina patients after removal of failed items (SAQ-CAN)

Hypothesised scale/item	Component/factor loadings			
	Factor 1	Factor 2	Factor 3	Factor 4
Dressing	0.767			
Walking indoors	0.566			
Showering	0.806			
Walking more than a block		0.631		
Running or jogging		0.888		
Lifting or moving heavy objects		0.779		
Participate in strenuous sports		0.858		
Symptoms of Angina during strenuous activities				0.525
Frequency of symptoms				0.656
Frequency of use of medication				0.481
Satisfaction that everything being done			0.796	
Satisfaction with doctor’s explanation			0.759	
Overall satisfaction with treatment			0.889	
Interference with enjoyment of life?				0.733
Feelings about symptoms persistent				0.705
Worry about heart attach/death				0.494

*SAQ* Seattle Angina Questionnaire, Factor 1: indoor physical functioning, Factor 2: outdoor physical functioning, Factor 3: treatment experience, Factor 4: angina symptoms burden

Table 5 describes the internal consistency and reliability of the 16-item SAQ-CAN in comparison with the original 19-item SAQ. SAQ-CAN items had a good level of item total correlation with the remainder of their scale (0.46–0.86) and exceeded the accepted standard of the midrange of 0.4–0.8 [29]. The average levels of item-total correlation exceeded those for the original SAQ (0.19–0.80). The Cronbach’s alpha values for the four dimensions of the 16-item SAQ ranged between 0.77 and 0.90, exceeding the Cronbach’s alpha values for the 5 subscales of the 19-item. Similarly, the 16-item SAQ had a higher intra-class correlation coefficient compared to the original SAQ, showing that it had greater reproducibility.

**Table 5:** Internal consistency and reliability of the SAQ-CAN and original SAQ

Instrument/scale	items	Items mean (SD)	Item-total correlation range	n	Cronbach’s alpha	Intra-class correlation coefficient (ICC)	Test retest ICC
*SAQ-CAN*							
Indoor physical functioning	3	14.27 (1.65)	0.56–0.67	4052	0.77	0.76	0.56
Outdoor physical functioning	4	14.29 (6.54)	0.70–0.86	4052	0.90	0.90	0.54
Angina symptoms burden	6	24.10 (5.50)	0.46–0.72	4052	0.82	0.72	0.48
Treatment experience	3	12.95 (2.48)	0.74–0.84	4052	0.89	0.89	0.64
*Original SAQ*							
Physical limitations	9	36.42 (9.34)	0.40–0.80	4052	0.89	0.86	0.59
Angina stability	1	3.84 (1.19)	-	4052	–	–	0.37
Angina frequency	2	9.90 (2.32)	0.49	4052	0.63	0.53	0.47
Treatment satisfaction	4	17.99 (2.77)	0.19–0.77	4052	0.77	0.72	0.62
Disease perception	3	10.37 (3.03)	0.50–0.67	4052	0.76	0.72	0.48

*SAQ-CAN* Seattle Angina Questionnaire Canadian version, *SAQ* Seattle Angina Questionnaire

Table 6 describes the measure of change using a paired t-test, effect size and SRM. The paired t-test showed that the longitudinal sample tended to have better functioning (*p* < 0.001) for all domains. Only treatment experience has a smaller effect size, while angina symptoms burden had a moderate effect size, and indoor physical functioning has a large effect size [30].

**Table 6:** Mean (95% confidence limits) baseline and 12-month SAQ-CAN and SAQ, effect size (ES), standardized response means (SRM)

PRO Domain	Baseline	12-month	*p* value	ES	SRM
*SAQ-CAN*					
Indoor physical functioning	75.57 (75.09–76.05)	89.53 (89.09–89.97)	< 0.001	1.42	1.30
Outdoor PHYSICAL FUNCTIONING	52.29 (50.77–53.82)	60.18 (58.83–61.53)	< 0.001	0.26	0.23
Angina symptoms burden	69.76 (68.77–70.74)	81.03 (80.23–81.83)	< 0.001	0.59	0.53
Treatment experience	83.36 (82.41–84.31)	86.38 (85.44–87.32)	< 0.001	0.15	0.15
*Original SAQ*					
Physical limitation	61.63 (60.67–62.58)	67.35 (66.50–68.20)	< 0.001	0.30	0.28
Angina stability	71.24 (69.87–72.62)	75.59 (74.31–76.87)	< 0.001	0.15	0.12
Angina frequency	78.73 (77.63–79.83)	88.67 (87.85–89.48)	< 0.001	0.48	0.42
Treatment satisfaction	82.47 (81.71–83.23)	85.82 (85.07–86.56)	< 0.001	0.21	0.29
Disease perception	61.78 (60.61–62.95)	76.47 (75.52–77.42)	< 0.001	0.65	0.58

There was a substantial improvement in functioning from baseline to 1 year. Response mean was smallest for treatment experience and outdoor physical functioning, but indoor physical functioning had the highest response mean.

Table 7 describes the association between the subscales of the SAQ-CAN, SAQ, HADS, and EQ5D VAS. The indoor and outdoor physical functioning subscales showed a significantly strong correlation with the physical limitation subscale of the SAQ. Angina symptoms burden of the SAQ-CAN was strongly correlated with angina symptoms and angina frequency subscales of the SAQ, while the treatment experience subscale of the SAQ-CAN was strongly correlated with the treatment satisfaction subscale of the SAQ. The SAQ-CAN subscales exhibited a moderate correlation with the EQ5D VAS, but weak correlations with depression and anxiety subscales of the HADs. The polyserial correlation of the SAQ-CAN subscale with the EQ5D subscale showed a moderate negative correlation (see Table 8).

**Table 7:** Correlation between SAQ-CAN and other measures

Instrument	SAQ-CAN				Original SAQ					Other measures		
	IPF	OPF	ASB	TRE	PL	AS	AF	TS	DP	EQ5D_VAS	HADS_D	HADS_A
IPF	1											
OPF	0.415	1										
ASB	0.384	0.469	1									
TRE	0.201	0.202	0.477	1								
PL	0.619	0.958	0.534	0.237	1							
AS	0.238	0.321	0.688	0.337	0.357	1						
AF	0.314	0.369	0.842	0.358	0.426	0.475	1					
TS	0.221	0.216	0.496	0.953	0.255	0.318	0.388	1				
DP	0.363	0.444	0.901	0.459	0.504	0.493	0.578	0.478	1			
EQ5D_VAS	0.293	0.298	0.394	0.281	0.347	0.259	0.265	0.282	0.412	1		
HADS_D	− 0.061	− 0.016	− 0.067	− 0.059	− 0.034	− 0.015	0.038	− 0.063	− 0.085	− 0.068	1	
HADS_A	0.055	0.053	0.143	0.100	0.060	0.094	0.083	0.102	0.156	0.115	0.684	1

*IPF* indoor physical functioning, *OP* outdoor physical functioning, *ASB* angina symptoms burden, *TRE* treatment experience, *PL* SAQ physical limitations, *AF* SAQ angina frequency, *AS* SAQ angina stability, *TS* SAQ treatment satisfaction, *DP* SAQ disease perception, *EQ5D_VAS* EQ5D visual analogue scale, *HADS_D* hospital anxiety and depression scale (HADS) depression subscale, *HADS_A* HADS anxiety subscale. All correlations are significant at either *p* < 0.01 or *p* < 0.05

**Table 8:** Polyserial correlation with the SAQ-CAN subscale, and EQ-5D subscales

	IPF	OPF	ASB	TRE
Mobility	− 0.400	− 0.435	− 0.526	− 0.301
Selfcare	− 0.423	− 0.289	− 0.360	− 0.199
Usual	− 0.378	− 0.457	− 0.584	− 0.261
Pain	− 0.313	− 0.353	− 0.644	− 0.349
Anxiety	− 0.268	− 0.238	− 0.479	− 0.339

*IPF* indoor physical functioning, *OPF* outdoor physical functioning, *ASB* angina symptoms burden, *TRE* treatment experience

## Discussion

This study evaluated the psychometric properties of the SAQ in a Canadian sample of patients with stable angina. The analysis revealed that the original factorial structure of the SAQ was not valid in our sample and resulted in the removal of three redundant items with a negligible contribution to the clinically meaningful dimensions. The resulting measure is the SAQ-CAN, which comprised of 16-items that aggregates into four subscales with excellent validity, reliability, and responsiveness. Unlike the 19-item SAQ comprising 5 subscales, the SAQ-CAN items aggregate into four subscales: namely indoor physical functioning, outdoor physical functioning, angina symptoms/burden, and treatment experience subscales. These findings are consistent with previous studies on the validation of SAQ in other populations where different number and types of subscales emerged [9, 11]. For example, the validation of the SAQ in a UK population of patients with stable angina resulted in a similar 16-item measure (SAQ-UK) with three subscales [9, 11]. Furthermore, another unique feature of the SAQ-CAN is its delineation of the physical functioning subscale into two separate subscales (indoor physical functioning and outdoor physical functioning subscales) in the SAQ-CAN. In contrast, the items of the “angina stability” and “angina frequency” subscales of the original SAQ constitute the angina symptoms/burden subscale of the SAQ-CAN. These differences are consistent with findings from other validation studies of the SAQ. For example, Kimble et al. [9] also reported the division of the physical limitation subscale into two separate factors including ‘limitation in activities with middle to high exertional requirements’ and ‘limitation in activities with low exertional requirements’ in women with chronic stable angina [9]. Similarly, the translation and validation of the Farsi version of the SAQ yielded a five-factor solution with subscales that were not identical to the original SAQ subscales [10]. This highlights the need for preliminary validation and adaptation of the measure in each population before its deployment in clinical care.

A major strength of this study is its investigation of both construct and factorial validity, reliability, and responsiveness of the SAQ-CAN. Although SAQ is a widely-used measure, its factorial validity has not been replicated in any other study. A possible explanation for this limitation may be attributed to the factorial structure and subscale composition of the SAQ, which included a subscale with a single item (angina frequency). The findings of this study will further facilitate the interpretation of the SAQ-CAN’s scores and changes in those scores over time. This study is not without its limitations. First, floor and ceiling effects in the items could result in difficulty discriminating between the functioning of individuals within the lower or upper range of the scale. There is floor effect in four items; “running or jogging”, “lifting or moving heavy objects”, “participate in strenuous sports” and “feelings about symptoms persistent”. Second, our assessment of the test–retest reliability of the SAQ was based on data collected between 1 year-interval. Test–retest reliability is usually assessed over much shorter periods than in this study, usually producing reliability estimates that are much closer to those derived from internal consistency tests. Our future research will seek to validate these findings in a Canadian prospective cohort study where the SAQ-CAN can be administered within a shorter interval to confirm its responsiveness. Third, the validation of the SAQ-CAN, which is a subset of the original 19-item SAQ using a sample of patients who completed the original 19-item SAQ, suggests that comparisons of strength of correlation between each measure and other measures are not entirely independent. This might lead to biased estimates of correlations and consequently influence conclusions about the validity of the SAQ-CAN. Fourth, the validation of the SAQ-CAN in this study was based on secondary analyses of the population based on data of patients with chronic stable angina who completed the SAQ along with other important measures in the APPROACH registry. Future research will seek to replicate these findings in a prospective longitudinal study of individuals with stable angina. Fifth, we did not investigate a split-sample approach for conducting EFA and CFA in our sample despite having a fairly large sample. Future research will seek to replicate this factorial structure of the SAQ-CAN in an independent sample of stable angina patients. Finally, our validation of the SAQ-CAN relies on classical test theory approaches, which are known for their shortcomings [29]. Future research will examine the use of modern classical test theory approaches (i.e., item response theory) to further study the psychometric properties of the SAQ-CAN.

In conclusion, this study provides evidence for the measurement properties of a Canadian version of the SAQ. The SAQ-CAN is recommended as a patient-reported outcome measure for use in clinical trials and observational studies to assess health outcomes and the effectiveness of interventions in Canadians with coronary artery disease.

## Data Availability

The datasets used in this study are not publicly available but, researchers who fulfill the criteria for access, as determined and approved by the University of Calgary Conjoint Health Research Ethics Board, can have access to the data.

## Abbreviations

SAQ: Seattle Angina Questionnaire
APPROACH: Alberta Provincial Project for Outcome Assessment in Coronary Heart disease
EFA: Exploratory factor analysis
CFA: Confirmatory factor analysis
RMSEA: Root mean square error of approximation
CFI: Comparative fit index
HADS: Hospital Anxiety and Depression Scales
EQ5D: Euro quality of life 5-dimension
EQ5D VAS: Euro quality of life 5-dimension visual analogue scale
SAQ-CAN: Seattle Angina Questionnaire Canadian version (SAQ-CAN)
CAD: Coronary artery disease
HRQOL: Health-related quality of life
HADS_A: Hospital anxiety and depression scales-anxiety symptoms
HADS_D: Hospital anxiety and depression scales-depression symptoms;
MI: Myocardial infarction
BMI: Body mass index
SD: Standard deviation
IPF: Indoor physical functioning
OPF: Outdoor physical functioning
ASB: Angina symptoms and burden
TRE: Treatment experience
ICC: Intra-class correlation coefficient
PRO: Patient reported outcomes
PL: Physical limitations
AF: Angina frequency
AS: Angina stability
TS: Treatment satisfaction
DP: Disease perception
SAQ-UK: Seattle Angina Questionnaire in a UK population
